# A neonatal case report of branchiooculofacial syndrome caused by a novel mutation in the TFAP2A gene and literature review

**DOI:** 10.1097/MD.0000000000034962

**Published:** 2023-11-03

**Authors:** Fangmei Luo, Meiling Lu, Lu Zhao, Ping Zhou

**Affiliations:** a Jinan University First Affiliated Hospital, Guangzhou, China; b Jinan University-affiliated Shenzhen Baoan Women’s and Children’s Hospital, Shenzhen, China.

**Keywords:** branchiooculofacial syndrome, neonate, TFAP2A gene

## Abstract

**Rationale:**

Branchiooculofacial syndrome (BOFS) is a rare autosomal dominant disorder with a diverse clinical phenotype. To summarise the clinical characteristics and genetic variations of neonatal-onset BOFS through a case study and literature review.

**Patient concerns:**

A preterm neonate with a very low birth weight, born at a gestational age of 29^+3^ weeks, exhibited cosmetic abnormalities at a postmenstrual age of 34^+6^ weeks, including microcleft lip, high arched palate, curved upper lip, low ear position, and ocular hypertelorism. Hence, a genetic test on peripheral blood was carried out.

**Diagnoses:**

The genetic testing showed a heterozygous variant of c.724G > A (p.Glu242Lys) in the exon 4 region of the TFAP2A (transcription factor AP-2-α) gene in the short arm of chromosome 6. BOFS was confirmed based on clinical appearance and the genetic result.

**Interventions:**

The patient underwent solely cleft lip repair at the age of 6 months with no further intervention.

**Outcomes:**

The infant shows normal growth and development at 1 year of age and subsequent follow-up.

**Lessons:**

The characteristic facial features, branchial skin defects, and ocular anomalies are the main clinical manifestations of BOFS with neonatal onset, but the diverse clinical phenotype and variable genetic variants pose certain challenges for clinical diagnosis.

## 1. Introduction

Branchiooculofacial syndrome (BOFS) is a rare autosomal dominant disorder with a diverse clinical phenotype that can present with branchial skin defects, ocular abnormalities, characteristic facial anomalies, as well as ectopic thymus, subscale cysts, renal and dental abnormalities.^[1]^ Milunsky found in 2008 that TFAP2A gene mutations are linked to BOFS, and that multiple symptoms can result from the same locus.^[1]^ Few cases of neonatal BOFS have been reported. In order to evaluate the clinical phenotypic traits of neonatal BOFS caused by TFAP2A gene mutations, we retrospectively analyzed the clinical data of one instance of neonatal-onset BOFS admitted to the hospital and reviewed the pertinent literatures.

## 2. Case report

**Figure 1.**
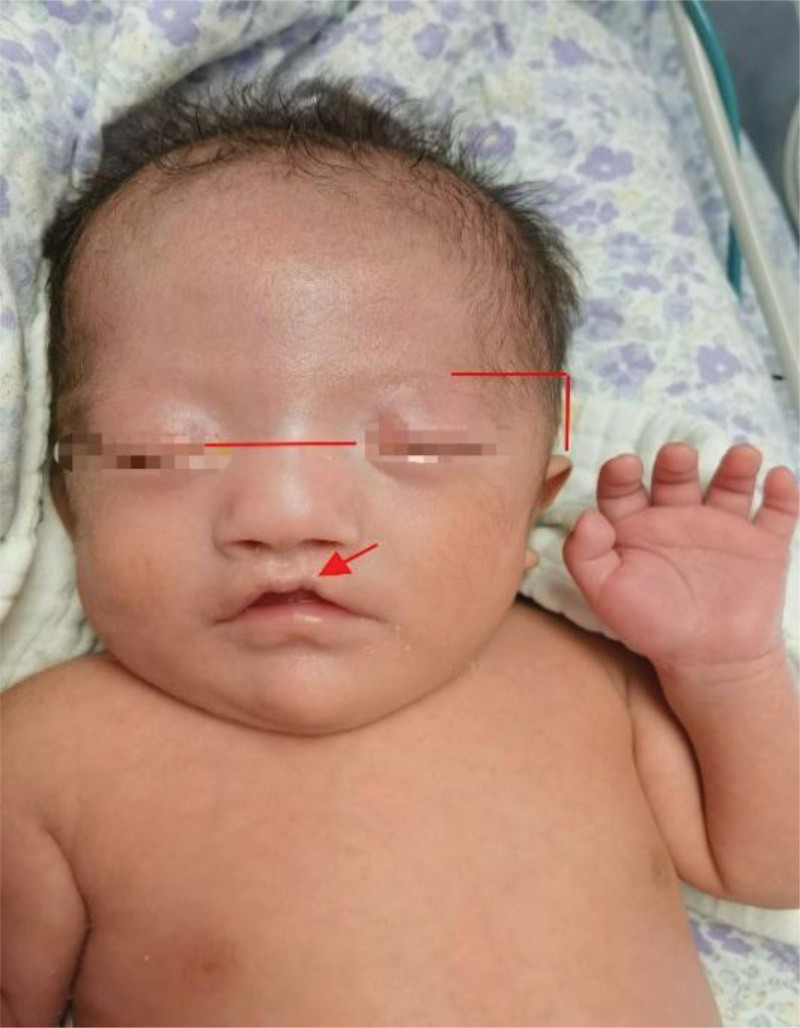
Picture of the facial features of the neonatal branchiooculofacial syndrome (BOFS) case at postmenstrual age (PMA) 34^+6^ wk: microcleft lip, ocular hypertelorism, curved upper lip, wide nasal bridge and low ear position.

An Asian female infant was admitted to the NICU with the chief complaint of birth weight of 1140 g and shortness of breath for 12 minutes after birth. She was G1P1, and single-course antenatal steroids and magnesium sulfate were used. She was delivered by emergency cesarean section at 29^+3^ weeks gestation due to severe maternal pre-eclampsia, without asphyxia. Her birth weight was 1.14 kg with a length of 39 cm and a head circumference of 23 cm. Because of extreme prematurity and neonatal respiratory distress syndrome, the infant received continuous invasive mechanical ventilation for 6 days immediately after birth, with no significant external abnormalities on routine physical examination. She then received NIPPV and nCPAP for 32 days because of bronchopulmonary dysplasia. When the respiratory support was changed to high-flow nasal cannula oxygen at postmenstrual age 34^+6^ weeks, the physician noted a discontinuity in the red border of the upper lip and what appeared to be a scar, which was diagnosed by the stomatologist as a microcleft lip. The presence of a high palatal arch, low ear position, ocular hypertelorism, wide nasal bridge, and curved upper lip were also noted (Fig. 1). A rapidly growing hemangioma on the skin of the right thigh was treated with oral propranolol and topical timolol; other abdominal, extremity, and neurological physical examinations were unremarkable. The parents were not consanguineous and there were no obvious clinical manifestations of BOFS. During follow-up after discharge, the infant developed an occipital subcutaneous cyst at 3 months of age and a lacrimal duct obstruction at 5 months of age, which was explored by lacrimal flushing, and the cleft lip repair was performed at 6 months of age. She is now 1 year old, and her body growth and neurological development are age-appropriate.

**Figure 2.**
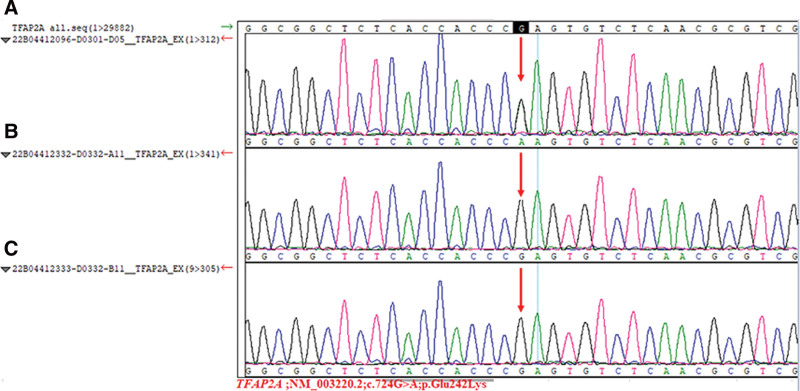
Results of genetic testing and parental Sanger validation in the neonatal BOFS case. The variant c.724G > A (p.Glu242Lys) was found in the TFAP2A gene on chromosome 6 in case (A), and the same variant was not found in the father (B) and mother (C).

Laboratory tests: complete blood count, liver and renal function, electrolytes, blood gas, thyroid function, and TORCH were normal. Ultrasound of the urinary tract showed a 3 mm separation of the left renal pelvis, and ultrasound of the heart, abdomen, and cranium showed no abnormalities. The hearing test was passed in both ears, and the retinopathy screening showed incomplete retinal vascularization bilaterally. On postnatal day 49, genetic testing was conducted at the UW Medical Laboratory in Shenzhen. Using microarray capture high-throughput sequencing, a heterozygous c.724G > A mutation was discovered in the protein-coding region of exon 4 of the TFAP2A gene (OMIM No. 113620) on chromosome 6 (chr6:10404781). Genetic informatics analysis of this locus variant revealed a glutamate-to-lysine substitution at position 242 of the TFAP2A protein (p.Glu242Lys). This locus variant has been reported as a pathogenic variant of BOFS.^[2]^ Sanger validation of the parental genes showed that neither had the same variants (Fig. 2). This gene variant was classified as a suspected pathogenic variant according to ACMG guidelines (Fig. 2).

## 3. Literature review

The PubMed, Web of Science, and Embase databases were searched using the keywords “BOFS” and “neonate or newborn.” From the time the database was established until April 2023, a total of 12 papers, including 16 cases with complete medical information, were searched for the diagnosis of BOFS in the neonatal period,^[3–14]^ plus the case reported, making a total of 17 cases. The 17 cases included 5 males and 12 females, 7 preterm and 10 term infants. The main clinical manifestations were: cleft lip in 16 cases (94.1%), low ear position in 14 cases (82.3%), branchial skin defects in 13 cases (76.5%), wide nasal bridge in 9 cases (52.9%), posteriorly rotated ears in 9 cases (52.9%), microphthalmia in 8 cases and ocular coloboma in 8 cases (47.0%), ocular hypertelorism, nasolacrimal duct stenosis/atresia, and high palatal arch in 7 cases each (41.7%), cleft palate, renal anomalies, and ectopic thymus in 5 cases each (29.4%). There was a family history of BOFS in 7 cases (Table 1). 10 of the 17 children underwent genetic testing and all were found to have different mutation loci in the TFAP2A gene, as shown in Table 1. In terms of follow-up and prognosis, 8 infants underwent repair of skin defects in the neck; one had spontaneous healing of the skin defect in the neck; 5 had excision of the gill cleft in the neck with a pathological report of the ectopic thymus; 6 had repair of cleft lip and palate; and 3 had hearing impairment with hearing aids. Most of the children had age-appropriate growth and development, with the exception of 1 case who died of renal failure due to renal dysplasia.

**Table 1:** Clinical data and genetic variant information from 17 neonates with BOFS.

Case No.	Gender	BW kg	GA *w*	Ocular anomalies			Craniofacial features							Branchial skin defects	Other anomalies	Genotype	Follow-up and prognosis
				Microph-thalmia	Nasolacrimal Duct stenosis	Ocular coloboma	Cleft lip	Cleft palate	High palatal arch	Wide nose bridge	Ocular hypertel-orism	Low ear position	Rotating ear				
1*	M	2.4	39	+	−	+	−	−	+	+	−	+	−	−	Upward sloping lids, small jaw	−	Skin repair of the neck, 4 yr; hearing loss in the left ear
2*	F	1.76	34	+			+	−	+	−	−	−	−	+	Recessed nasal tip, small jaw	−	Normal growth and development
3	F	3.1	38	−	−	+	+	−	+	−	−	+	−	+	Small jaw	Heterozygous c.629T > A	NR
4	F	NR	Term	−	−	−	+	+	−	+	+	−	−	−	Polycystic kidney, wide nasal tip	(c.767C > T, p.A256V)	Cleft lip, palate, and nasal tip repair, 1 yr; neck cyst removal, 4 yr; retinal detachment treatment, 5 yr
5	F	NR	37	−	−	−	+	−	−	+	−	+	+	+	Upward sloping lids, renal anomalies, ectopic thymus	R254Q mutation	Posterior auricular sinus and ectopic thymus surgery, 4 mo; cleft lip and nasal repair, 9 mo
6*	M	1.65	34	−	−	+	+	−	−	−	−	+	−	−	Renal anomaly	−	Died 6 h after birth due to renal failure
7	M	2.95	38	+	+	+	+	−	−	+	−	+	+	+	Ectopic thymus	Heterozygous c.731T > C (p.L244P)	Normal neuromotor development, 14 mo
8	F	2.91	Term	−	−	−	+	−	−	+	+	+	+	+	Polycystic kidney	missEnse p.A256V (c.767 C > T)	Refuse to treat self-healing of skin defects in the neck
9	F	1.66	38	+	+	−	+	−	+	−	+	+	+	+	Ectopic thymus and renal anomaly	−	Hearing aid, 4 mo; skin and ear repair, 1yr
10	M	3.2	−	−	+	−	+	−	−	−	+	+	+	+	Ectopic thymus	−	Gill-cleft sinus remove, 2 mo; hearing aid in the left ear, 6 mo; good prognosis,7 yr
11*	M	1.4	32	+	+	+	+	+	−	−	−	+	−	+	Small jaw	NM_003220.2:c.699A > C	NR
12*	F	NR	Preterm	−	+	−	+	+	−	−	+	+	+	+	Small jaw	NM_003220.2:c.699A > C	NR
13*	F	3.8	39	−	+	−	+	+	+	+	+	+	+	+	−	Heterozygous c.740C.A [p.Ser247X]	Cleft lip and palate repair, 4 mo; skin defect repair, 5 yr; orthodontics, 10 yr
14*	F	2.85	39	+	−	+	+	−	+	+	−	−	−	+	−	Heterozygous missense H384Y	Orbital cyst treatment
15	F	1.15	32	+	+	+	+	+	−	+	−	+	+	+	−	−	Cleft lip, branchial skin defects, and cleft palate repair, 9 mo; skin repair of left ear, 13 mo
16	F	3.6	Term	+	−	+	+	−	−	−	−	+	+	+	Ectopic thymus	−	Cleft lip repair, 15 mo; branchial skin defects repair, 7 yr; blindness
17	F	1.14	29	−	−	−	+	−	+	+	+	+	−	−	Occipital scalp cyst	Heterozygous c.724G > A	Cleft lip repair, 6 mo normal at 1-yr follow-up

BW = birth weight, GA = gestation age, NR = not reported.

* Confirmed case of branchiooculofacial syndrome (BOFS) in a first-degree relative of the child.

## 4. Discussion

We report a case of BOFS in a premature infant with a typical clinical presentation and confirmed genetic result and present the first systematic summary of the clinical features and genetic variation characteristic of neonatal-onset BOFS cases.

In the early 1980s, Lee et al^[15]^ first defined branchial skin defects, ocular anomalies, and characteristic facial features as a new gill slit syndrome, and in 1987, Fujimoto et al^[16]^ introduced the term “branchioocuofacial syndrome.” Milunsky et al^[2]^ analyzed 41 cases of BOFS in 30 families and concluded that the following conditions should be present to confirm the diagnosis of BOFS: branchial skin defects; ocular anomalies (microphthalmia, strabismus, coloboma, cataract, nasolacrimal duct stenosis/atresia, etc); characteristic facial manifestations (cleft lip, pseudo labial cleft with or without cleft palate, wide nasal bridge, flattened nasal tip, high palatal arch, ocular hypertelorism, low and posteriorly rotated ears, etc). The diagnosis is confirmed by any 2 of the above major presentations plus one of the following: ectopic thymus; first-degree relative with BOFS. Among the 17 cases of neonatal BOFS summarized in this paper, characteristic facial features (100%), branchial skin defects (76.5%), and ocular anomalies (76.5%) were the 3 most common clinical manifestations, with almost half of the cases (47%) having all 3 major clinical manifestations. The ectopic thymus is an additional diagnosis, with the majority of non-neonatal BOFS cases diagnosed in the published literature having an ectopic thymus, and the neonatal case in this paper also had 5 cases of the ectopic thymus. The very preterm infant in this paper required prolonged respiratory support after birth due to respiratory distress syndrome and bronchopulmonary dysplasia, and the lips and face were partially covered with tape and ventilator tubing until the infant was found to have characteristic facial features at postmenstual age 34^+6^ weeks. Although there were no branchial skin defects or ocular anomalies, the subsequent presence of hemangiomas, skin cysts in the occipital region of the head, obstructed lacrimal ducts and abnormal genetic results supported the diagnosis of BOFS. This suggests that the clinical manifestations of BOFS are not always apparent in early life and may develop progressively over different periods of life, requiring greater awareness by clinicians and careful physical examination to find the abnormal presentation early.

The gene responsible for BOFS, TFAP2A, is located in the P24.3 region of chromosome 6, contains 437 amino acids, and encodes the protein transcription factor AP-2-α, which is associated with gene regulation of the eye, ear, face, trunk, and neural tube during embryonic development.^[8]^ When mutated, the TFAP2A gene can lead to abnormal development of these organs. The TFAP2A gene was first linked to the clinical presentation of BOFS in 2008.^[1]^ Among BOFS cases, 95% had TFAP2A gene coding sequence alterations in genetic testing, mainly involving exon 4 (82%), exon 5 (14%), and the remaining exons accounting for 4%. No clear genotype-phenotype correspondence has been found^[2]^: cases with the same genetic variant have significantly different phenotypes both within and between families, while different genetic variants, such as missense, shift, and splice variants and more complex rearrangements can lead to similar phenotypes. In this paper, the TFAP2A gene variants were present in all 10 genetically tested cases, and the TFAP2A mutation loci differed between families, mainly by missense and shift mutations, resulting in corresponding amino acid changes and thus a different clinical phenotype.

BOFS is a rare disease of unknown prevalence that is inherited in an autosomal dominant manner with almost complete epistasis. Children with BOFS have a 50% chance of inheriting the causative variant and require good risk assessment and genetic counseling. 50% to 60% of BOFS cases result from the de novo causative variant, while 40% to 50% of cases are inherited from the parents, a proportion consistent with this paper, where 41% of children (7 cases) were found to have first-degree relatives with BOFS, including 1 infant with the same TFAP2A mutation (NM_003220.2:c.699A > C) and clinical presentation as his mother (cases 11 and 12). The TFAP2A gene of the preterm infant in this paper was detected with the c.724G > A heterozygous mutation, but no identical mutation was found in the parental Sanger test, suggesting the possibility of a spontaneous mutation.

In this literature review, 7 cases were diagnosed with BOFS based on clinical presentation alone, which may have been misdiagnosed due to a lack of genetic testing to confirm the diagnosis. This is because BOFS shares similar skin abnormalities and mode of inheritance with a gill slit ear and kidney syndrome. However, gill-cleft ear and kidney syndrome are primarily characterized by urinary tract damage and kidney abnormalities, whereas BOFS is characterized by facial and ocular abnormalities. It also needs to be distinguished from CHARGE syndrome and 22q11.2 deletion syndrome. Both can present with eye, ear, and orofacial clefts, but CHARGE syndrome has no skin defects and no BOFS facial features, and deletion syndrome often has heart defects without BOFS facial features. An accurate diagnosis requires a clinical phenotype combined with genetic testing.

For treatment, children with BOFS are followed up by a multidisciplinary team (including craniofacial specialists, plastic surgeons, otolaryngologists, and speech therapists) for specialized assessment and appropriate surgery based on the duration and severity of the child main symptoms. Of the 14 infants for whom follow-up information was available, 9 underwent various procedures, including gill cleft repair and gill sinus excision, cleft lip and palate repair, skin cyst excision and skin defect repair, ear and nose repair, nasolacrimal duct exploration and ectopic thymus surgery. The overall prognosis of BOFS is good, with improvement in appearance and function with surgery, plastic surgery, and hearing aids, but in some cases, multiple fetal deaths have occurred within the family,^[11]^ with genes suggesting the same mutation in the mother and fetus. Whether this miscarriage was caused by a simple mutation in the TFAP2A gene or combined with other abnormalities needs further investigation. Another neonatal case developed renal failure 6 hours after birth due to renal dysplasia and died from multiple organ failure.^[7]^ More data is needed to further investigate whether mutations in the BOFS gene lead to severe phenotypes.

By describing a case of a unique TFAP2A gene mutation that caused neonatal BOFS and reviewing the pertinent literature, we discovered that the majority of neonatal BOFS cases presented with cleft lip, branchial skin abnormalities, and eye deformities, as well as multiple genetic variations. A molecular diagnosis can be made by genetic testing when children with BOFS are confirmed to show clinical signs but do not meet clinical diagnostic criteria. Following the discovery of genetic mutations, families should be offered genetic counseling and the chance for prenatal diagnostics.

## Author contributions

**Writing – original draft:** Fangmei Luo, Meiling Lu.

**Writing – review & editing:** Lu Zhao, Ping Zhou.
